# Map of ubiquitin-like post-translational modifications in chronic lymphocytic leukemia. Role of p53 lysine 120 NEDDylation

**DOI:** 10.1038/s41375-021-01184-7

**Published:** 2021-05-08

**Authors:** Sara Bravo-Navas, Lucrecia Yáñez, Íñigo Romón, Montserrat Briz, Juan José Domínguez-García, Carlos Pipaón

**Affiliations:** Laboratorio de Hematología Molecular, Servicio de Hematología, Hospital Universitario Marqués de Valdecilla-IDIVAL, Santander, Spain

**Keywords:** Chronic lymphocytic leukaemia, Apoptosis

## To the Editor:

Chronic lymphocytic leukemia (CLL) has focused a strong research effort over the last years due to its high incidence, that will likely increase due to the progressive ageing of the population, and the lack of truly curative therapies. Genome-wide screenings in CLL patients revealed a group of mutations in genes covering divergent cellular pathways [1]. Unfortunately, the mutations found did not affect to more than 13% of the patients at its best. New genome-wide studies widen the search of mutations to cover untranslated and regulatory regions of the genome [2].

At the sight of all of these absolutely necessary but enormously dispersed genomic and gene expression data, some researchers have started to point to post-translational mechanisms as the unifying defect explaining the common cellular characteristics of CLL. Noteworthy, several preclinical studies have tested the use of inhibitors of some of these post-translational modifications. This is the case of MLN4924-pevonedistat, an investigational inhibitor of protein NEDDylation that is being tested in clinical trials for the treatment of different neoplasias (https://clinicaltrials.gov/ct2/results?term=MLN4924&Search=Search).

We wanted to investigate the real extension of the NEDDylation-related alterations in B-CLL cells. Our hypothesis is that a transversal process like this post-translational modification may be able to provide a unifying explanation to the wide array of alterations related to the development of CLL, thus providing molecular support to the use of NEDDylation inhibitors in the treatment of this disease.

Current whole-cell profiling techniques do not allow the specific identification of NEDDylated proteins, so we used a method of immunoaffinity enrichment of proteins containing a K-GG remnant left by ubiquitin or ubiquitin-like (UBL) peptides after digestion with trypsin. The peptides thus purified were then identified by mass spectrometry. The application of this method over samples of B-CLL cells and CD19+ cells obtained from healthy donors allowed us to have a wide view of the differentially ubiquitin or UBL-modified proteins in this pathology. However, the diGly remnant left on ubiquitinated peptides after tryptic digestion is shared by ubiquitin, NEDD8 and ISG15 modifications. In order to discriminate those peptides originally conjugated to NEDD8 from those modified by ubiquitination or other UBLs, we analyzed the same B-CLL samples but treated with the NEDDylation inhibitor MLN4924.

**Fig. 1:**
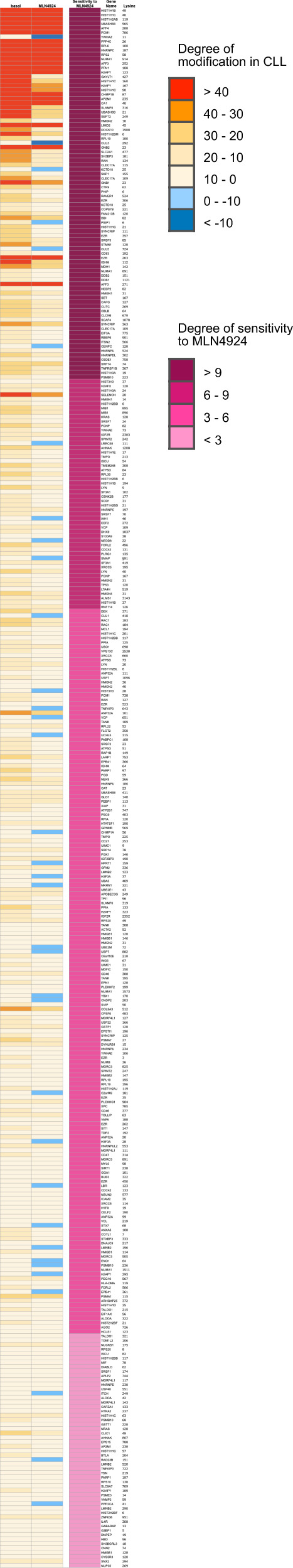
List of the 353 over-modified and MLN4924-sensitive lysines found in CLL B cells. On the left, the heatmap shows the fold change in the modification state of the lysines in CLL B cells compared to healthy donor cells at basal state and after treatment with 0.25 µM MLN4924. The sites are sorted by their sensitivity to MLN4924, expressed as the difference in the fold change between the basal and MLN4924 conditions.

The procedure identified a total of 3384 differentially modified peptides in CLL corresponding to 1746 proteins. 651 site modifications were unreported at the date of the screening. Of those K-GG remnant modifications, 2142 (63%) corresponded to a ≥2.5-fold increase in B-CLL cells with respect to the healthy controls, while only 401 (12%) were reduced ≥2.5-fold. Of those 2142 augmented modifications, 353 were notably reverted by the incubation with MLN4924 (threshold 2.5-fold) (Fig. 1). Noteworthy, 76% of these sites were not detected by the UbiSite antibody in the work by Akimov et al. [3].

Overall, MLN4924 affected the amount of the K-GG remnant in 1666 sites of 1008 proteins (threshold 2.5-fold); post-translational modifications induced by MLN4924 were found in 609 proteins, while modifications impaired by the inhibitor were found in 399 proteins. Interestingly, 37 proteins displayed modifications of both types.

Although these data suggest an increment of NEDDylated proteins in CLL, we found no differences in the NEDD8 present in the serum of a group of patients when compared to that of healthy donors (Supplementary figure 1). However, the expression of the genes coding for the E1 (NAE1) and the two E2’s (UBE3F and UBE2M) specific of the NEDDylation pathway were higher in peripheral mononuclear cells from the patients (Supplemental Fig. 2).

Our screening showed that the modification status of several members of the NEDDylation cascade were increased in CLL and sensitive to the action of the NEDDylation inhibitor (Supplementary Table 1). It also unveiled the altered post-translational modification of a group of proteins related to the ubiquitination machinery, many of them sensitive to the action of MLN4924 (Supplementary Table 2). We found alterations in the post-translational modification of many proteins involved in pathways previously described as relevant to the pathogenesis of CLL (Supplementary Tables 3–7). These pathways include DNA repair, RNA processing, and the NF-kB pathways, previously reported to be modulated by post-translational modifications, although our screening unveiled novel proteins and lysine targets, as well as a their sensitivity to MLN4924 treatment. This is the case of the NF-kB pathway where, surprisingly, no change in IkB modification was detected in our screening [4, 5]. Rather, upstream regulatory members of the pathway showed these kind of changes (see Supplementary Table 7). Among them, TANK stood out for the over-modification of three of its lysines in CLL, that could be partially reverted by MLN4924.

Structural proteins associated with phenotypical changes in CLL cells also were aberrantly modified. This is the case of proteins of the chromatin and the cytoskeleton (Supplementary Tables 4 and 5). Vimentin levels in CLL B cells have been inversely associated with the apparition of smudge cell in peripheral blood smear preparations [6, 7] and with a worse prognosis [8]. Our screening unveiled an increase in post-translational modifications of vimentin and other cytosqueleton proteins and treatment with MLN4924 reverted those modifications along with a reduction in the amount of smudge cells (Supplemental Fig. 3). Although a more profound study is needed, these results suggest a negative effect of NEDDylation over the function of vimentin, and a role of post-translational modifications in the molecular basis of a characteristic cellular manifestation of CLL.

Post-translational modifications of p53 play a key role in its function as tumor supressor and many of them have been described [9, 10]. Our screening did not detect modifications in the C-terminal lysines 370, 372, and 373 of p53 in CLL. By contrast, we found a strong modification of lysine 120, that was sensitive to the treatment with MLN4924 (Supplementary Table 8). Ubiquitination and acetylation of this lysine have been previously described [11, 12]. To test whether this residue of p53 could also be NEDDylated, we generated the mutant K120R and cotransfected it in cells along with MDM2 and NEDD8. Compared to wild-type p53, this mutant showed a reduced sensitivity to NUB1L, a protein that specifically targets NEDDylated proteins to the proteasome [13], suggesting a reduction in its NEDDylation level (Supplementary Fig. 4).

**Fig. 2:**
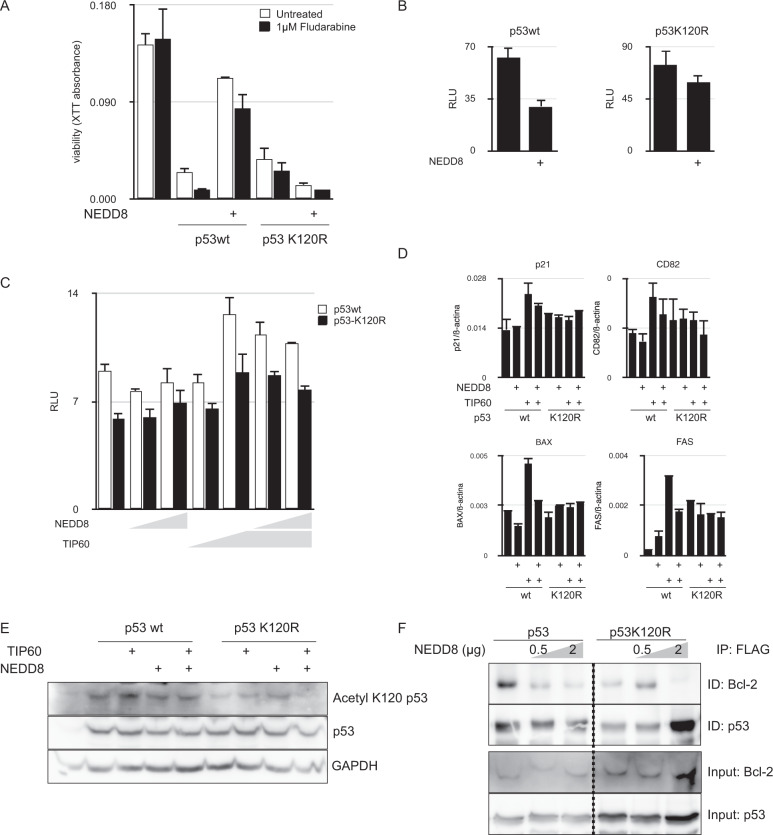
Molecular study of the effect of K120 NEDDylation over p53 function as tumor suppressor. **A** NEDDylation of p53 at lysine 120 affects its transactivation potential and the cell viability. XTT cell viability assay of MEC1 cells nucleofected with p53 wt or p53 K120R in the presence or absence of MDM2 and NEDD8. The effect of fludarabine in MEC1 viability in each condition is also depicted. **B** The luciferase reporter vector pG13Luc was used to measure the transactivation potential of p53 wt or the K120R mutant in the presence or absence of NEDD8. **C** NEDD8 interferes with the acetylation of p53 at lysine 120. Luciferase assay of pG13Luc cotransfected with p53 wt or the K120R mutant along with MDM2, the acetylase TIP60 and increasing amounts of NEDD8. **D** Analysis by RT-qPCR or the expression of four p53 target genes in HEK293T cells transfected with expression plasmids for the indicated proteins. **E** Western blot analysis of the acetylation of p53 at lysine 120 in HEK293T cells transfected with expression plasmids for the indicated proteins. **F** NEDDylation impairs p53 interaction with Bcl-2. Coimmunoprecipitation of FLAG-tagged p53 wt or K120R mutant with Bcl-2 in the presence of increasing amounts of NEDD8. Whole-cell extracts of p53 wt- or K120R mutant-transfected HEK293T cells were precipitated with an anti-FLAG antibody and the associated Bcl-2 was revealed by western blot.

We then investigated the role of NEDDylation of lysine 120 on the function of p53. To this end, we used the LLC cell line MEC1 that is defective in p53. Transfection of wild-type p53 in MEC1 cells induced a reduction in viability that was partially reverted by cotransfection with MDM2 and NEDD8. K120R mutant also induced a reduction in MEC1 cells viability, although NEDD8 cotransfection did not revert it (Fig. 2A). These experiments suggest that p53 NEDDylation in lysine 120 reduces its capacity to act as a tumor suppressor.

We next investigated the molecular basis of this impairment of p53 function. First, we tested the effect of lysine 120 NEDDylation on the transactivation potential of p53. In our transient transfection experiments, K120R mutation did not significantly affect p53 transactivation capacity but rather incremented it. However, when NEDDylation was induced by MDM2 and NEDD8 overexpression, p53 experimented a strong reduction in its transactivation potential while for p53K120R the inhibition was milder (Fig. 2B). Acetylation of lysine 120 has been demonstrated to be essential for p53 to develop its full transactivation potential. Indeed, cotransfection of TIP60, one of the acetylases known to modify lysine 120 of p53, induced an increase in the activation of a p53 reporter construct (Fig. 2C), as well as in the expression of p53 target genes (Fig. 2D). We then wanted to study whether NEDDylation could be interfering with lysine 120 acetylation. Cotransfection of MDM2 and NEDD8 impaired TIP60 enhancement of p53 transactivation potential strongly when lysine 120 was intact (Fig. 2C, D). A western blot analysis with an antibody specific for K120-acetylated p53 demonstrated that NEDDylation reduced the acetylation of lysine 120 (Fig. 2E).

Forced ubiquitination of both p53 forms induced a similar reduction in their transactivation capacity (Supplementary Fig. 5A). However, this reduction in p53 transactivation activity was due to a destabilization of the protein that does not occur with its NEDDylation (Supplementary Fig. 5B).

Acetylation of lysine 120 has also been related to the transcriptional-independent induction of apoptosis by p53, through the modulation of its interaction with Bcl-2 [14, 15]. When overexpressed in HEK293 cells, p53 interacted with Bcl-2, as we could demonstrate in coimmunoprecipitation experiments. However, the amount of Bcl-2 pulled down by p53 was reduced in a dose dependent manner when MDM2 and NEDD8 were transfected along (Fig. 2F). Accordingly, K120R mutation of p53 greatly impaired its interaction with Bcl-2.

This work provides an overall view of the UBL modifications in chronic lymphocytic leukemia, that brings the idea that an alteration in such transversal processes could be an underlying link among the variety of pathways involved in this pathology, giving rationale for the use of MLN4924 in its treatment.

## Supplementary information

Supplemental figures

Supplemental tables
