# Supplementary material for: Map of ubiquitin-like post-translational modifications in chronic lymphocytic leukemia. Role of p53 lysine 120 NEDDylation
Source: Leukemia. 2021 May 8;35(12):3568–72. doi: 10.1038/s41375-021-01184-7 (PMC8632665; doi:10.1038/s41375-021-01184-7)
Supplement: Supplementary file 1 — Supplemental figures [file 41375_2021_1184_MOESM1_ESM.pdf]

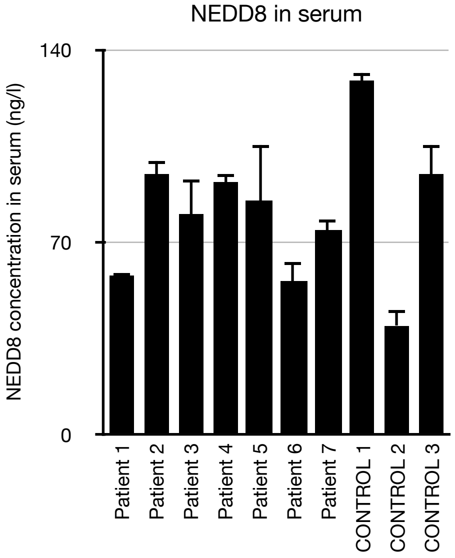

Supplemental Figure 1. NEDD8 determination in the blood serum of CLL patients.
Serum from blood samples of patients and healthy donors were obtained by clotting and centrifugation, after informed consent. NEDD8 was determined in the sample serums in duplicate using an ELISA assay.

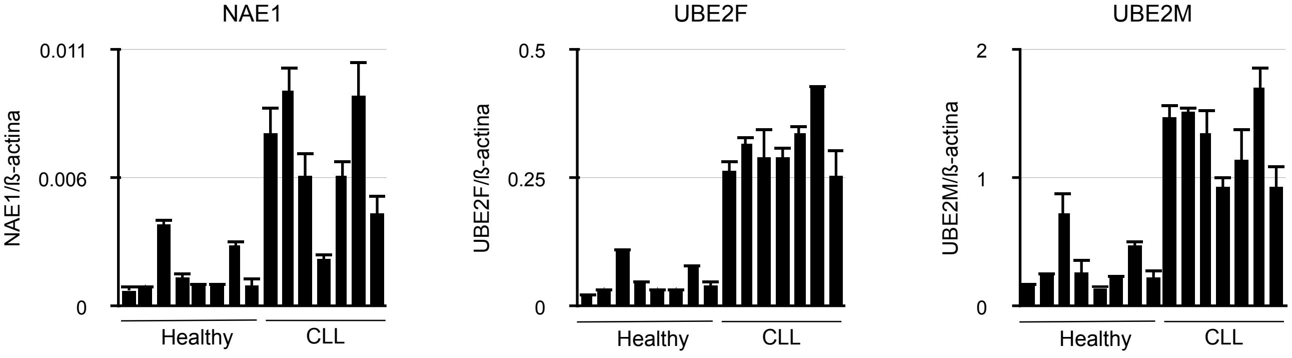

Supplemental Figure 2. Expression of the E1 and E2'2 genes in CLL
RT-qPCR analysis of the mRNA expression of NAE1, UBE2F and UBE2M in peripheral blood mononuclear cells obtained from 7 patients of CLL and 8 healthy donors. All three genes show a higher expression in B-CLL cells than in healthy mononuclear cells (Control vs. CLL p=0.001 for NAE1 and p<0.001 for UBE2F and UBE2M, Fisher-Pitman test).

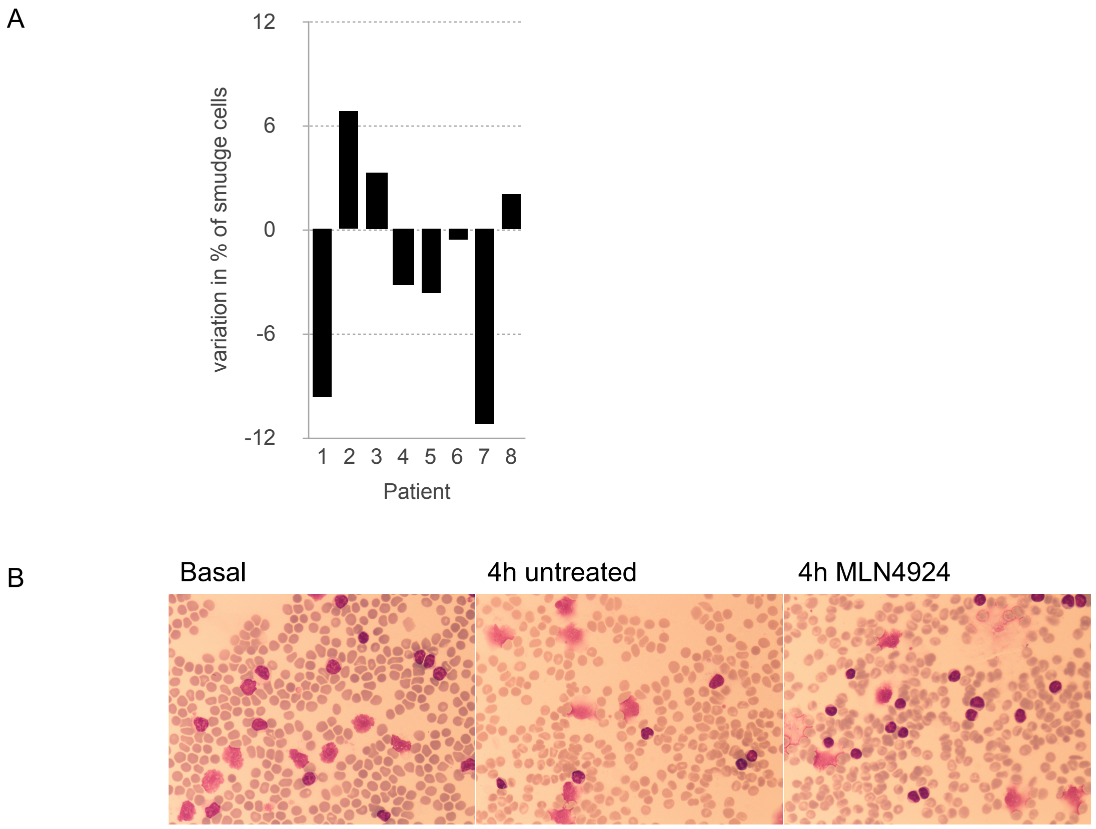

Supplemental Figure 3. Effect of MLN4924 over the fragility of CLL B-cells.
Samples of peripheral blood obtained from 8 CLL patients were incubated for 4 hours in the presence or absence of MLN4924. Smear preparations from each condition were analyzed for the quantification of the number of smudge cells. A. The difference between the percentage of smudge cells in MLN4924-treated versus non treated in 8 different samples from CLL patients is represented. B. Typical microscopic fields obtained in the analysis of smear preparations from patient number 1.

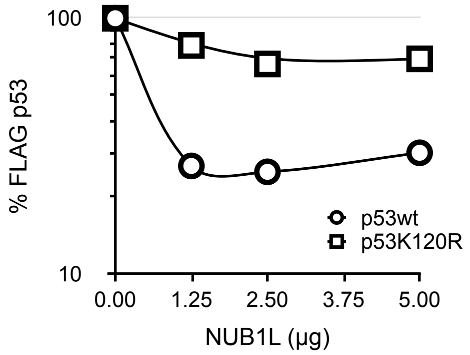

Supplemental Figure 4. K120R mutation desensitizes p53 to NUB1L .
In the presence of MDM2 and NEDD8, p53 wild type is more sensitive to the degradation mediated by NUB1L than the K120R mutant. HEK293T cells were transfected with MDM2, NEDD8 and either FLAG-tagged p53 wt or the K120R mutant along with increasing amounts of NUB1L. The amount of p53 were analyzed by western blot and the intensity of the bands obtained quantified. The figure depicts the percentage of the corresponding p53 form in the presence of increasing amounts of NUB1L relative to that in the absence of it.

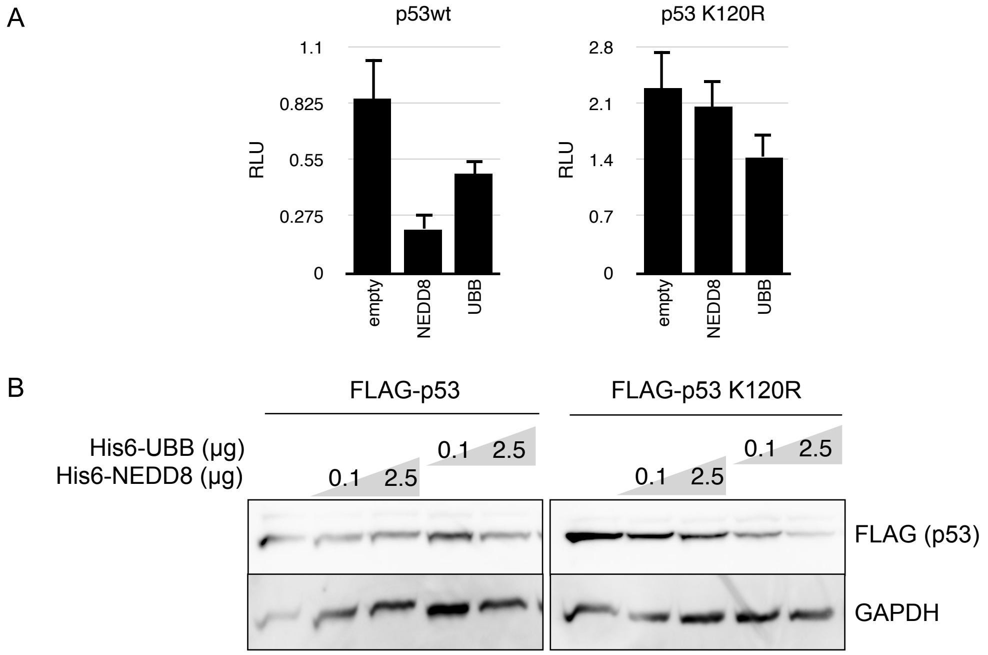

Supplemental Figure 5. Ubiquitination destabilizes p53 while its NEDDylation does not.
A. Luciferase assay of pG13Luc cotransfected with p53 wt or the K120R mutant along with MDM2 and NEDD8 or ubiquitin as indicated. B. Western blot analysis of the amount of p53 wt or K120R mutant in the presence of MDM2 and increasing amounts of NEDD8 or ubiquitin.
