# Supplementary material for: Map of ubiquitin-like post-translational modifications in chronic lymphocytic leukemia. Role of p53 lysine 120 NEDDylation
Source: Leukemia. 2021 May 8;35(12):3568–72. doi: 10.1038/s41375-021-01184-7 (PMC8632665; doi:10.1038/s41375-021-01184-7)
Supplement: Supplementary file 2 — Supplemental tables [file 41375_2021_1184_MOESM2_ESM.pdf]

TABLES

For each modified lysine, the mean of the fold change in CLL samples treated either with vehicle (DMSO) or with 0.25 µM MLN4924 with respect to untreated healthy donors samples, is shown.

Table 1. Modification of proteins related to the NEDDylation pathway in CLL.

Protein	Mean DMSO CLL vs Control	Mean MLN4924 CLL vs Control	Site	Peptide
**CUL1**	**4.0**	**-2.0**	410	FINNNAVTK*MAQSSSK
**CUL3**	**3.4**	**-16.5**	292	NGK*TEDLGCMYK
**CUL5**	**8.2**	**-4.8**	724	TQEAIIQIMK*M#R
**NAE1**	**-2.0**	**-27.3**	338	GTIPDMIADSGK*YIK
**NAE1**	**-2.8**	**-18.9**	12	LLKEQK*YDR
**UBA3**	**6.5**	**1.9**	409	SPAITATLEGK*NR
**UBE2M**	**2.0**	**-2.4**	92	VGQGYPHDPPK*VK
**UBE2M**	**2.6**	**-1.7**	72	LVICPDEGFYK*SGK
**DCUN1D1**	**-0.4**	**-5.2**	143	AQIPK*MEQELKEPGR
**DCUN1D1**	**-1.9**	**-3.3**	63	K*KLEQLYNR
**DDB1**	**14.0**	**2.1**	1121	MQEVVANLQYDDGSGMK*R
**COPS7B**	**14.9**	**0.3**	221	TLK*ATASSSAQEMEQQLAER
**NEDD8**	**1.8**	**-12.5**	54	QMNDEK*TAADYK
**NEDD8**	**2.0**	**-8.2**	6	VK*TLTGKEIEIDIEPTDKVER
**NEDD8**	**2.5**	**-4.2**	22	TLTGKEIEIDIEPTDK*VER
**NEDD8**	**1.7**	**-4.0**	48	LIYSGK*QMNDEK
**NEDD8**	**-1.5**	**-5.4**	11	VK*TLTGK

Table 2. Modification of proteins related to the ubiquitination machinery in CLL.

Protein	Mean DMSO CLL vs Control	Mean MLN4924 CLL vs Control	Site	Peptide	Involvement in pathways relevant in CLL
**RBCK1**	**-5.3**	**-14.4**	174	GPLEPGPPK*PGVPQEPGR	p53, NF-kB
**MIB1**	**20.1**	**11.4**	895	AAVLFQPCGHMCACENCANLMK*K	Notch, NF-kB
**MIB1**	**20.1**	**11.4**	896	AAVLFQPCGHMCACENCANLMKK*	
**PCNP**	**27.3**	**18.8**	82	K*ASAISIK	MAPK, PI3K/AKT/mTOR
**PCNP**	**10.0**	**3.7**	167	NIK*SHLGNVHDQDN	
**RNF185**	**-4.7**	**-12.4**	105	EK*TPPRPQGQRPEPENR	Wnt
**ZNF313**	**19.9**	**13.9**	126	ATIK*DASLQPR	p21
**XIAP**	**12.9**	**8.0**	31	LK*TFANFPSGSPVSASTLAR	Apoptosis
**MKRN1**	**4.0**	**-0.5**	321	SAK*QFESK	Wnt, p53, p21
**UBE2E1**	**17.7**	**13.1**	43	NSK*LLSTSAK	Involved in AML
**TRIM22**	**-6.0**	**-9.3**	265	SESWTLK*KPK	PI3K/AKT/mTOR
**TRAF2**	**-0.9**	**-4.6**	176	APCCGADVK*AHHEVCPK	NF-kB and JNK
**TRAF2**	**-1.5**	**-4.6**	27	TLLGTK*LEAK	
**TRAF2**	**-5.7**	**-8.6**	119	GTLK*EYESCHEGR	
**ITCH**	**2.8**	**-0.0**	249	VSGNNSPSLSNGGFK*PSRPPRPSRPPPPTPR	Role in CLL, Jun
**cIAP2**	**0.0**	**-2.6**	231	HFPK*CPFIENQLQDTSR	Role in CLL, NF-kB
**USP22**	**11.0**	**6.9**	166	K*ITSNCTIGLR	Potential oncogene
**RBBP6**	**18.4**	**8.6**	901	NIGSNYPEK*LSAR	p53
**Cbl-b**	**6.4**	**4.0**	881	RLPGENVK*TNR	BCR signaling
**Cbl-b**	**27.4**	**17.1**	64	LCQNPK*LQLK	
**SKP1A**	**23.1**	**4.5**	155	K*ENQWCEEK	Cell cycle
**SKP1A**	**3.9**	**1.5**	128	TVANMIK*GK	
**USP7**	**4.2**	**-0.1**	882	KLYYQQLK*MK	p53, Notch, NF-kB, Wnt
**USP7**	**13.2**	**7.7**	1096	YTYLEK*AIK	
**UCHL5**	**4.8**	**-0.4**	315	TLAEHQQLIPLVEK*AK	Wnt, TGFß
**USP48**	**5.6**	**2.7**	551	LTVK*ALCK	NF-kB, DNA repair
**ataxin-3**	**-22.4**	**-27.0**	117	SFICNYK*EHWFTVR	DNA repair, cytoskeleton

Table 3. Modification of proteins related to the DNA repair machinery in CLL.

Protein	Mean DMSO CLL vs Control	Mean MLN4924 CLL vs Control	Site	Peptide
**DDB2**	**13.4**	**1.6**	151	DKPTFIK*GIGAGGSITGLK
**H2AX**	**19.5**	**10.6**	128	KTSATVGPK*APSGGKK
**Ku80**	**7.7**	**1.4**	195	LGGHGPSFPLK*GITEQQK
**Ku80**	**7.6**	**2.0**	660	FNNFLK*ALQEK
**XPC**	**2.1**	**-3.0**	508	KDPSLPAASSSSSSSK*R
**XPC**	**9.0**	**5.2**	785	K*LDIDCVQAI
**Ku70**	**9.0**	**5.6**	114	NIYVLQELDNPGAK*R
**MORF4L1**	**10.6**	**6.5**	127	ANQEQYAEGK*MR
**MORF4L1**	**9.1**	**5.5**	111	YVDTNLQK*QR
**MORF4L1**	**8.3**	**5.4**	117	ELQK*ANQEQYAEGK
**MORF4L1**	**9.5**	**6.6**	143	TSGLQQK*NVEVK
**RAD23B**	**2.6**	**-0.0**	151	QEKPAEK*PAETPVATSPTATDSTSGDSSR

Table 4. Modification of histones and DNA binding proteins in CLL.

Protein	Mean DMSO CLL vs Control	Mean MLN4924 CLL vs Control	Site	Peptide
**H1B**	**911.8**	**516.9**	49	ATGPPVSELITK*AVAASK
**H1B**	**8.6**	**1.1**	194	SPAKPK*AVKPK
**H1B**	**11.7**	**5.6**	37	K*ATGPPVSELITK
**H1B**	**3.4**	**-0.5**	17	SETAPAETATPAPVEK*SPAK
**H1C**	**-90.8**	**-398.1**	17	AAPPAEK*APVK
**H1C**	**58.7**	**28.8**	160	K*PAAATVTK
**H1C**	**21.9**	**8.2**	21	PAAPAAAPPAEKAPVK*K
**H1C**	**17.5**	**9.3**	159	KAK*KPAAATV
**H1C**	**12.2**	**6.3**	201	AAKPK*VVKPK
**H1C**	**724.4**	**415.1**	46	KASGPPVSELITK*AVAASK
**H1C**	**17.6**	**14.8**	63	SGVSLAALK*K
**H1C**	**60.4**	**32.4**	90	SLVSK*GTLVQTK
**H1C**	**-6.4**	**-10.3**	63	SLAALK*K
**H1C**	**8.5**	**5.8**	97	GTLVQTK*GTGASGSFK
**H1D**	**7.3**	**4.2**	35	RK*ASGPPVSELI
**H1E**	**10.8**	**3.0**	17	SETAPAAPAAPAPAEK*TPVK
**H1X**	**3.8**	**0.3**	19	TTAEGMAK*K
**H1X**	**1.9**	**-0.8**	23	KVTK*AGGSAALSPSK
**H2AE**	**13.4**	**9.5**	119	VTIAQGGVLPNIQAVLLPK*KTESHHKT
**H2AE**	**1.8**	**-1.5**	100	LLGK*VTIAQGGVLPNIQAV
**H2AFY**	**569.1**	**537.5**	123	GK*LEAIITPPPAK
**H2AFY**	**65.0**	**36.1**	167	QGEVSK*AASADSTTEGTPADGFTVLSTK
**H2AFY**	**1.0**	**-3.7**	332	QTAAQLILK*AI
**H2AFY**	**11.3**	**6.9**	323	NGFPK*QTAAQLILK
**H2AFY**	**-12.7**	**-16.1**	117	ASGGVLPNIHPELLAKK*
**H2AFY**	**2.9**	**-0.2**	295	TVK*NCLALADDKK
**H2AFY**	**10.2**	**7.6**	189	AASADSTTEGTPADGFTVLSTK*SLFLGQK
**H2B**	**9.8**	**4.0**	117	HAVSEGTK*AVTKYTSSK
**H2B**	**4.6**	**1.6**	117	HAVSEGTK*AVTK
**H2B**	**15.4**	**7.7**	6	PEPSK*SAPAPK
**H2B1D**	**16.0**	**7.3**	6	PEPTK*SAPAPK
**H2B1D**	**12.1**	**4.8**	21	KAVTK*AQK
**H2B1L**	**11.4**	**5.9**	6	PELAK*SAPAPK
**H2B1M**	**45.0**	**23.2**	6	PEPVK*SAPVPK

**H2B2F**	**5.5**	**2.9**	6	PDPAK*SAPAPK
**H2B2F**	**11.3**	**8.3**	21	AVTK*VQK
**H3**	**20.4**	**11.4**	19	K*QLATK*AAR
**H3**	**13.1**	**4.2**	24	KQLATK*AAR
**H3F3A**	**4.0**	**-0.6**	37	PSTGGVK*KPHR
**H3F3A**	**3.5**	**-0.2**	28	K*SAPSTGGVK
**HIST1H2AE**	**292.8**	**142.6**	119	QGGVLPNIQAVLLPK*K*
**HIST1H2AE**	**20.0**	**14.2**	120	VTIAQGGVLPNIQAVLLPK*K*TESH
**HIST3H3**	**1.2**	**-4.4**	37	SAPATGGVK*KPHR
**HIST3H3**	**4.5**	**-1.0**	28	K*SAPATGGVK
**HIST3H3**	**9.4**	**0.5**	37	TGGVK*KPHR
**HMGN2**	**12.3**	**6.0**	31	LSAK*PAPPKPEPKPK
**HMGN2**	**9.4**	**4.0**	36	LSAKPAPPK*PEPKPK
**HMGN2**	**9.4**	**4.0**	40	LSAKPAPPKPEPK*PK
**HMGN2**	**8.8**	**4.5**	31	LSAK*PAPPKPEPK
**HMGN2**	**29.8**	**6.9**	16	AK*VKDEPQR
**HMGN4**	**21.1**	**14.9**	31	LSAK*PAPPKPEPR
**NUCKS**	**17.3**	**14.3**	175	LK*ATVTPSPVK
**ZNF638**	**13.9**	**11.4**	951	K*AAESMVK

Table 5. Modification of proteins of the cytoskeleton in CLL.

Protein	Mean DMSO CLL vs Control	Mean MLN4924 CLL vs Control	Site	Peptide
**ACTA2**	**4.9**	**0.5**	52	HQGVMVGMGQK*DSYVGDEAQSK*R
**CAP1**	**-12.3**	**-15.2**	126	GSK*LFNHLSAVSESI
**Cofilin-1**	**-31.1**	**-34.3**	112	FWAPESAPLK*SK
**EPB41**	**7.4**	**2.4**	366	ELEEK*VMELHK
**EPB41**	**2.7**	**-0.4**	361	LAPNQTK*ELEEK
**Ezrin**	**22.7**	**9.3**	357	LQDYEEK*TK
**Ezrin**	**9.5**	**4.2**	523	RITEAEK*NER
**Ezrin**	**6.5**	**2.9**	450	AK*EAQDDLVK
**Ezrin**	**7.9**	**4.1**	35	QLFDQVVK*TIGLR
**Ezrin**	**28.3**	**13.3**	306	RKPDTIEVQQMK*AQAR
**Ezrin**	**73.8**	**61.3**	263	K*APDFVFYAPR
**Ezrin**	**8.6**	**4.8**	262	FVIKPIDK*K
**Ezrin**	**13.0**	**9.0**	3	PK*PINVR
**Lamin B1**	**-6.5**	**-30.0**	417	K*RVDVEESEASSSV
**Lamin B1**	**1.7**	**-4.7**	111	CK*AEHDQLLLNYAK
**Lamin B1**	**1.6**	**-3.8**	109	AKLQIELGK*CK
**Lamin B2**	**0.0**	**-4.8**	186	AQLAK*AEDGHAVAK
**Lamin B2**	**5.9**	**1.3**	123	LQIEIGK*LR
**Lamin B2**	**2.9**	**-0.3**	196	K*QLEKETLMR
**Lamin B2**	**6.8**	**4.1**	520	FTPK*YILR
**Lamin B2**	**6.1**	**3.5**	290	LDSAK*LSSDQNDK
**profilin 1**	**194.6**	**154.0**	108	TDK*TLVLLMGK
**STMN1**	**30.2**	**16.9**	128	DK*HIEEVR
**TUBB1**	**-35.3**	**-55.9**	324	MSTK*EVDQQLLSVQTR
**Vimentin**	**2.1**	**-3.0**	168	RQVDQLTNDK*AR
**Vinculin**	**7.8**	**4.4**	219	NSK*NQGIEEALK

Table 6. Modification of proteins of the RNA splicing machinery in CLL.

Protein	Mean DMSO CLL vs Control	Mean MLN4924 CLL vs Control	Site	Peptide
**SF3A1**	**8.6**	**1.1**	102	HKVSEFK*EGK
**SF3A1**	**16.8**	**10.4**	419	ASKPLPPAPAPDEYLVSPITGEK*IPASK
**SFRS3**	**12.3**	**7.2**	23	VYVGNLGNNGNK*TELER
**SFRS3**	**27.8**	**14.4**	85	VRVELSNGEK*R
**SFRS7**	**16.3**	**7.8**	24	VYVGNLGTGAGK*GELER
**SFRS7**	**13.0**	**5.7**	70	GLDGK*VICGSR
**SFRS15**	**11.7**	**1.5**	1078	GK*EKPEVTDR
**SF1**	**1.9**	**-1.9**	227	NILK*QGIETPEDQNDLRK
**SF2**	**4.9**	**2.1**	174	K*LDNTK*FR

Table 7. Modification of proteins of the NF-kB pathway in CLL.

Protein	Mean DMSO CLL vs Control	Mean MLN4924 CLL vs Control	Site	Peptide
**IKKß**	**3.6**	**7.6**	106	K*YLNQFENCCGLR
**NEMO**	**3.6**	**5.4**	344	LK*ASCQESAR
**TANK**	**9.0**	**3.8**	189	LNIPDTATETQCSVPIQCTDKTDK*QEALFK*PQAK
**TANK**	**7.5**	**3.2**	308	TTDK*TKPSNLVNTCIR
**TANK**	**7.9**	**3.7**	195	LNIPDTATETQCSVPIQCTDKTDKQEALFK*PQAK*DDINR
**TRAF2**	**-0.9**	**-4.6**	176	APCCGADVK*AHHEVCPK
**TRAF2**	**-1.5**	**-4.6**	27	TLLGTK*LEAK
**TRAF2**	**-5.7**	**-8.6**	119	GTLK*EYESCHEGR
**CD40**	**-36.2**	**-66.2**	267	QEPQEINFPDDLPGSNTAAPVQETLHGCQPVTQEDGK*ESR

Table 8. Modification of p53 and related proteins in CLL.

Protein	Mean DMSO CLL vs Control	Mean MLN4924 CLL vs Control	Site	Peptide
**p53**	**13.7**	**7.5**	120	LGFLHSGTAK*SVTCTYSPALNK
**USP7**	**13.2**	**7.7**	1096	YTYLEK*AIK
**USP7**	**4.2**	**-0.1**	882	KLYYQQLK*MK
**TP53RK**	**0.4**	**5.1**	40	FLSGLELVK*QGAEAR
